# Bioprospecting a Film-Forming System Loaded with *Eugenia uniflora* L. and *Tropaeolum majus* L. Leaf Extracts for Topical Application in Treating Skin Lesions

**DOI:** 10.3390/ph16081068

**Published:** 2023-07-27

**Authors:** Mariana Dalmagro, Mariana Moraes Pinc, Guilherme Donadel, Getulio Capello Tominc, Ezilda Jacomassi, Emerson Luiz Botelho Lourenço, Arquimedes Gasparotto Junior, André Giarola Boscarato, Salviano Tramontin Belettini, Odair Alberton, Inara Staub Prochnau, Reinaldo Aparecido Bariccatti, Rafael Menck de Almeida, Kelen Menezes Flores Rossi de Aguiar, Jaqueline Hoscheid

**Affiliations:** 1 Laboratory of Preclinical Research of Natural Products, Paranaense University, Umuarama 87502-210, Brazil; mariana.dal@edu.unipar.br (M.D.); mariana.pinc@edu.unipar.br (M.M.P.); donadel425@gmail.com (G.D.); getulio.tominc@edu.unipar.br (G.C.T.); ezilda@prof.unipar.br (E.J.); emerson@prof.unipar.br (E.L.B.L.); andreboscarato@prof.unipar.br (A.G.B.); salviano@prof.unipar.br (S.T.B.); odair@prof.unipar.br (O.A.); 2 Laboratory of Cardiovascular Pharmacology (LaFaC), Faculty of Health Sciences, Federal University of Grande Dourados, Dourados 79804-970, Brazil; arquimedesjunior@ufgd.edu.br; 3 School of Medicine and Life Sciences, Pontifical Catholic University of Paraná, Toledo 85902-532, Brazil; inara.prochnau@pucpr.br; 4 Center for Engineering and Exact Sciences, State University of Western Paraná, Toledo 85903-220, Brazil; reinaldo.bariccatti@unioeste.br; 5 Synthetica Research and Technical Analysis Ltda., Capela do Alto, São Paulo 18195-000, Brazil; rafaelmenck@synthetica.com.br; 6 Group of Polymers and Nanostructures, Federal Technological University of Paraná, Toledo 85902-490, Brazil; kelenaguiar@utfpr.edu.br

**Keywords:** polyvinyl alcohol, chaguinha, checkerboard, pitanga, polyvinylpyrrolidone

## Abstract

Natural products can be used as complements or as alternatives to synthetic drugs. *Eugenia uniflora* and *Tropaeolum majus* are natives of Brazil and have antimicrobial, anti-inflammatory, and antioxidant activities. This study aimed to develop a film-forming system (FFS) loaded with plant extracts with the potential for treating microbial infections. *E. uniflora* and *T. majus* leaf extracts were prepared and characterized, and the individual and combined antioxidant and antimicrobial activities were evaluated. The FFS was developed with different concentrations of polyvinylpyrrolidone (PVP) and polyvinyl alcohol (PVA) and analyzed for physicochemical characteristics. The combination of extracts showed a superior antioxidant effect compared to the individual extracts, justifying the use of the blend. FFS prepared with 4.5% PVA, 4.5% PVP, 7.81% *E. uniflora* extract, and 3.90% *T. majus* extract was adhesive, lacked scale formation, presented good malleability, and had a suitable pH for topical application. In addition, the viscosity at rest was satisfactory for maintaining stability; water solubility was adequate; skin permeation was low; and the antimicrobial effect was superior to that of the individual extracts. Therefore, the developed FFS is promising for the differentiated treatment of skin lesions through topical application.

## 1. Introduction

Administering drugs through the skin has several advantages, such as reduced side effects, constant and non-invasive drug delivery, and easy application. Conventional treatment consists of applying ointments, creams, and patches; however, these have several limitations [1,2]. Therefore, a novel alternative to conventional topical and transdermal formulations may be the film-forming system (FFS) [3].

FFS allows the delivery of drugs in the form of a polymeric solution, which produces a film, at the desired location [2]. With the formation of a thin film, the concentration of the drug increases, approaching the degree of supersaturation of the drug at the site. This phenomenon improves the thermodynamic activity of the formulation, disrupts the dermal barrier, and, consequently, reduces irritation and side effects [4].

Medicinal plants have long been used as sources for the development of drug formulations and modified delivery systems [5]. *Eugenia uniflora* L. is originally from the Atlantic Forest and is popularly known as pitangueira; it has high levels of phenolic derivatives in its fruits and leaves. According to the literature, *E. uniflora* has a highly complex chemical composition both in its fruits and leaves, including the presence of essential oils, sesquiterpenes, tannins, flavonoids, anthocyanins, saponins, mineral salts, and vitamin C [6]. These compounds have proven therapeutic activities, including, specifically, anti-inflammatory, antioxidant, analgesic, antidiabetic [7,8], hepatoprotective [9], and antifungal [10,11] activities.

*Tropaeolum majus* L. is distributed worldwide and is popularly known as nasturtium and chaguinha [12]. Several classes of secondary metabolites have been isolated and further identified in *T. majus* preparations. Glycosinolates, terpenoids, and flavonoids are the main groups of secondary metabolism of the plant, which are related to its pharmacological actions [13]. It exhibits antihypertensive [14], diuretic [15], and anti-inflammatory [16] activities, and it is used to treat asthma and constipation [12,17,18]. In addition, it is a source of antibacterial and antifungal agents [12,18,19].

Both extracts have already been used in topical formulations and showed no signs of cytotoxicity. A preclinical study by Donadel et al. [20] in a toxicity model evaluated the safety of a topical formulation containing *E. uniflora* leaf extract in rats for 14 days. The analyses were carried out in the vaginal region with high vascularity. No histological, haematological, or biochemical changes were found in the results obtained, proving the safety of using the extract under the evaluated conditions.

Both extracts have also already been used in skin lesion models through topical use, with the *T. majus* extract showing healing improvement in a wound model proposed by Correa et al. [21]. The study compared tissue restoration using two topical formulations, one carbopol-based gel and the other containing plant extract (10%). The wounds were evaluated at 7, 14, and 21 days, and after completion, it was found that the gel containing *T. majus* showed better healing process recovery.

Poly(vinyl alcohol) (PVA) is a synthetic biopolymer generated through the hydrolysis of polyvinyl acetate that was first prepared in 1924 by Hermann and Haehnel. Due to its solubility in water, it is used for several applications [22]. The PVA polymer has been widely used in the development of formulations due to its high mechanical resistance and elasticity, non-toxicity, and adhesiveness, favoring a better pharmacotechnical aspect [23].

Polyvinylpyrrolidone (PVP) is a water-soluble polymer widely used as an excipient for developing pharmaceutical formulations [24]. This polymer can act as a coating agent, binder, suspending agent, and solubilizer. In addition, it can be incorporated into traditional formulations and new controlled delivery systems due to its stabilizing, biocompatible, and biodegradable characteristics [25].

In this context, this study aimed to expand the findings on the application of biomaterials in the development of natural formulations through the bioprospecting of an FFS loaded with leaf extracts of *E. uniflora* and *T. majus*, which may be a differentiated treatment for topical infections.

## 2. Results and Discussion

This study characterized the soluble ethanolic fractions of *E. uniflora* (SEEU) and *T. majus* (SETM) extracts and evaluated their combined and isolated effects on microorganisms in topical infectious processes. We aimed to develop a colloidal FFS loaded with SEEU and SETM.

### 2.1. Characterization of SEEU and SETM

The phytochemical characterization of SEEU and SETM is presented in Table 1 and Table 2, respectively.

Because part of the extract may be composed of structural materials originating from primary metabolism and not directly related to biological activities [26], the preparation methodology was based on protein precipitation, a fact that tends to decrease the overall yield of the extractive process but increases the bioactive content, facilitating its binding on the target site. Additionally, ethyl alcohol is used as a solvent because it is accessible, obtained from a renewable source (sugarcane), classified as GRAS (generally recognized as safe), and is suitable for an approach to plant extract production [27].

In SEEU, flavonoids constitute the most representative class among the compounds identified, which is similar to the findings of Sobral-Souza et al. [28]. In this study, the authors point out that the extract of *E. uniflora* is a promising source of flavonoids, which have a cytoprotective effect due to their antioxidative capacity.

Astragalin (kaempferol-3-O-glucoside) belongs to the class of glycosylated flavonoids and is the primary compound in SETM, as reported elsewhere by Gasparotto Junior et al. [14]. In this research, the authors evaluated the antihypertensive activity of isoquercitrin and the hydroethanolic extract of *T. majus*. Phytochemical identification verified high levels of isoquercitrin and kaempferol glycoside in both the extract and the semi-purified fraction.

Flavonoids act as antioxidants in the inactivation of reactive oxygen and nitrogen species [29] and exhibit anti-inflammatory, immunomodulatory [26], and antimicrobial activities [30], showing broad pharmacological potential. The combined and individual antioxidative capacities of SEEU and SETM were measured using 2,2-diphenyl-1-picryhydrazyl (DPPH), ferric reducing ability of plasma (FRAP), and 2,2-azinobis(3-ethylbenzthiazoline-6-sulfonic acid) (ABTS^•+^) (Table 3). The theoretical value of the combination was estimated from the individual values and the proportions used to prepare the FFS.

The total amount of phenolic compounds and flavonoids directly influences the antioxidant ability; when the content of these bioactive compounds increases, the reducing potential tends to increase [31]. Phenol derivatives have antioxidant properties both at the beginning and during the oxidative process by neutralizing or scavenging free radicals [32], justifying the antioxidative capacity observed in this study, where several bioactive compounds of the phenolic class, such as quercetin, astragalin, and myricetin, were present during phytochemical identification. Furthermore, the scavenging potential of DPPH and FRAP radicals and the reducing potential of FRAP in SEEU and SETM were higher than those previously reported for *E. uniflora* [33] and *T. majus* [34].

During the combined antioxidant analysis of SEEU and SETM, an antagonistic effect for DPPH and synergistic effects for the FRAP and ABTS^•+^ methods were observed. The synergistic mechanism of the antioxidative capacity has not yet been elucidated due to the complex nature of the mixtures, mainly of plant extracts. However, some issues can be considered to justify this effect, namely, regeneration of the most effective antioxidant response, formation of stable intermolecular complexes, formation of phenolic products with more significant potential for reduction, and the occurrence of unexpected interactions between the compounds [35].

Oxidative stress impacts the inflammatory response; therefore, the presence of antioxidants can provide a favorable environment for the healing process [36]. Plant extracts with antioxidative capacity are promising for incorporation into auxiliary formulations for the healing process and tissue reconstitution. The combination of SEEU and SETM increased the antioxidant effect through FRAP and ABTS^•+^ mechanisms, justifying the use of the blend for the development of FFS.

The minimum inhibitory concentration (MIC) of SEEU and SETM (Table 4), individually and in combination, was evaluated using the checkerboard model.

The hydroalcoholic leaf extracts of *E. uniflora* had MIC of 12.5, 15.0, and 17.5 mg mL^−1^ for *Staphylococcus aureus*, *Escherichia coli*, and *Candida albicans*, respectively [31]; the results from this study show a similar range. Few studies have investigated the antimicrobial activity of *T. majus* leaf extract, and the MIC has not been evaluated [37]; however, antimicrobial activity against *S. aureus*, *E. coli*, *Pseudomonas aeruginosa*, and *C. albicans* was observed for both aqueous and ethanolic extracts.

The biological activities of extracts are directly influenced by the classes of compounds they contain [38]. Therefore, the combination of extracts exhibited increased antioxidant and antimicrobial potential against the microorganisms tested. Both SEEU and SETM had an inhibitory effect against the evaluated strains. However, because the Inhibitory Fractional Concentration Index (FICI) value was greater than 0.5, no synergistic effect of enhanced antimicrobial activity was observed. No antagonistic effect was observed (FICI > 4) for the combination. Therefore, considering the presence of a variety of bioactive compounds in SEEU and SETM, their association may exert complementary effects for the treatment of pathologies [39].

Based on the antioxidant and antimicrobial effects of SEEU and SETM, FFS loaded with SEEU and SETM was bioprospected.

### 2.2. Characterization of FFS

The experimental approach consisted of simultaneously evaluating the influence of the concentrations of PVP and PVA polymers in the system because these variables can influence each other, and their ideal values may be interdependent [40].

The organoleptic characteristics allowed for observing the formation of films with an adhesive character, regardless of the concentrations of PVA and PVP (Table 5). Adherence is an important aspect because it guarantees a prolonged period of contact between the formulation and the skin tissue, increasing the efficiency in delivering the active compounds to the site [41]. In addition, it reduces the risk of transfer of the active compounds to other individuals or clothes [3].

The control FFSs were transparent and had a drying time shorter than or close to that of the film loaded with SEEU and SETM, which showed a greenish-yellow color, characteristic of plant materials. Formulations 1 and 2 (F1 and F2, respectively) exhibited a scaly appearance, which can be attributed to the low concentration of PVA in the system. PVA is widely used owing to its high mechanical resistance and elasticity, non-toxicity, and bioadhesion, which constitute better pharmacotechnical parameters for the development of formulations [23]. The presence of scales in a formulation leads to detachment during movement; therefore, this would not be ideal for an optimized formulation.

**Figure A1:**
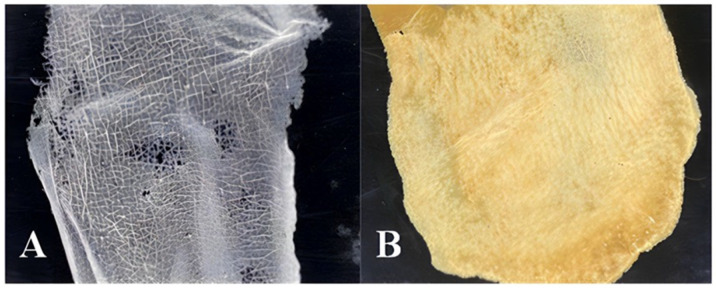
Organoleptic analysis of FFS. (**A**) = Control 4 (C4). (**B**) = Formulation 4 (F4).

The FFS formulations 3, 4, and 5 (F3, F4, and F5, respectively) showed an opaque, non-scaly, flexible, and non-sticky appearance (Figure A1), ensuring prolonged adhesion to the site and the release of active compounds for a longer period [42]. When evaluating the effect of the variables (PVA and PVP) on the drying time, no marked differences were observed between the FFS loaded with SEEU and SETM; however, F4 and F5 presented a drying time close and inferior to that of F3.

For physical–chemical characterization, the pH, volume delivered after each actuation, and viscosity of the FFS were evaluated (Table 6). The presence of extracts reduced the pH in all FFSs compared to that in the controls; however, the formulations maintained values between 4.37 and 5.3, similar to the pH of human skin, indicating suitability for topical application without discomfort [4,43].

The FFS was applied using a metered dose valve for delivering a fixed amount of product topically [3]. For each FFS, the volume was determined 10 times, and the observed standard deviation was acceptable. This is important because it ensures consistency in the delivered dose and, consequently, in the bioactive concentrations [4].

**Figure 1:**
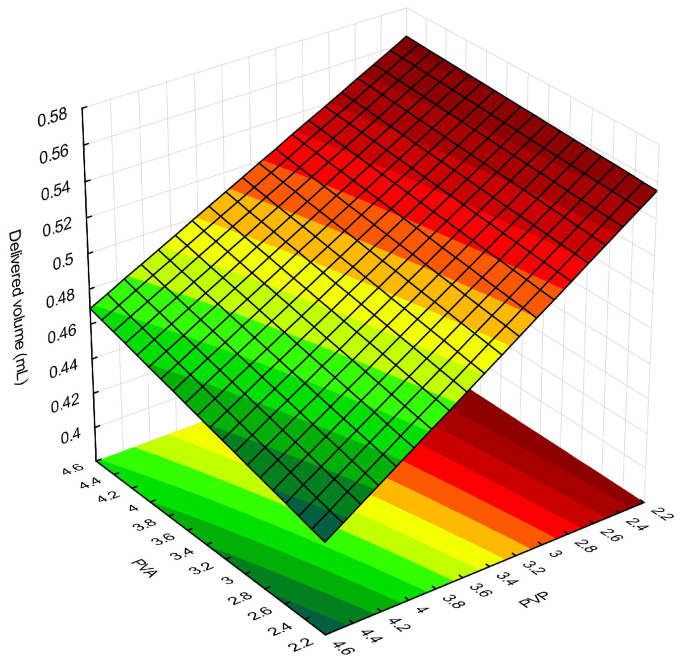
Response surface for the delivery volume of FFS loaded with SEEU and SETM as a function of polyvinylpyrrolidone (PVP) and polyvinyl alcohol (PVA) concentrations.

Delivery volumes of FFS loaded with SEEU and SETM ranged from 0.43 to 0.56 mL, with an increase in PVP concentration leading to a notable decrease in volume (Figure 1). PVP can increase viscosity [44] and consequently influence the delivered volume of the formulation because higher amounts of fluid formulations flow more easily [45].

**Figure 2:**
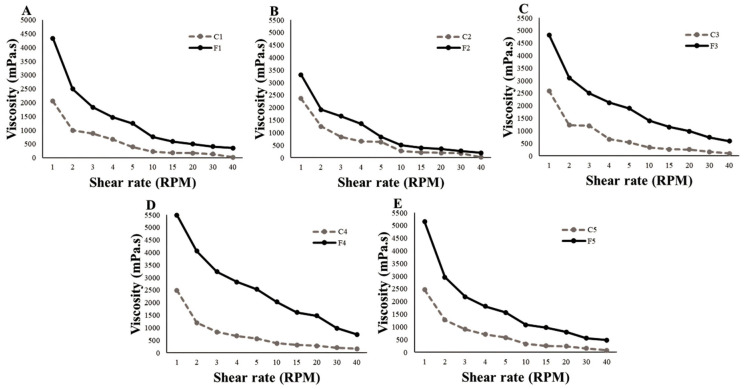
Rheogram of FFS control and loaded with SEEU and SETM. (**A**) = Formulation 1. (**B**) = Formulation 2. (**C**) = Formulation 3. (**D**) = Formulation 4. (**E**) = Formulation 5.

The viscosity and rheological behavior of formulations must be determined, as they are associated with product preparation, transport, storage, and shelf life [46,47]. The concentrations of PVA and PVP and the inclusion of SEEU and SETM in the FFS resulted in changes in viscosity and rheological behavior (Figure 2). This can be observed in the higher values of the initial viscosity (1 RPM) of systems F4 and F5, which contained a higher concentration of polymers.

An increase in the initial viscosity of all FFSs was observed when SEEU and SETM were added; high consistency indices at rest indicate that the system is structured. In general, when the shear force increased, the viscosity decreased; this is characteristic of formulations with pseudoplastic flow. When the shear rate increases, the polymeric structure is organized along the shear direction; therefore, the subsequent shear occurs more quickly and the apparent viscosity decreases [47].

Formulations with pseudoplastic flow allow for adequate delivery. Despite the high viscosity of the formulations at rest when an expulsion force is applied with high shear rates, the viscosity tends to decrease, which allows the formulation to flow easily out of the bottle dispenser during valve actuation, ensuring ease of application of the product by the patient [47].

### 2.3. Characterization of the Optimized FFS

**Figure 3:**
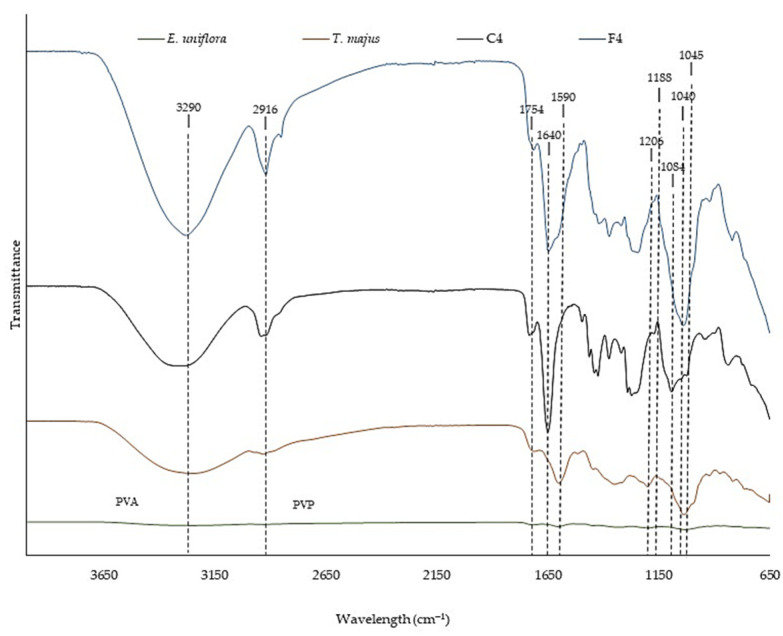
Fourier transform infrared (FTIR) spectrum of SEEU and SETM, control FFS (C4), and loaded with SEEU and SETM (F4).

The evaluation of the Fourier transform infrared (FTIR) spectra allows chemical analysis of the raw materials and the interactions between the components of a formulation [48]. Figure 3 shows the FTIR spectra of SEEU and SETM, control FFS (C4), and FFS loaded with SEEU and SETM (F4).

SEEU and SETM peak at 1754 cm^−1^, characteristic of elongation of the carbonyl group, and at 1590 cm^−1^, indicative of vibration of the aromatic rings [49] of phenolic derivatives. In addition, peaks at 1188 cm^−1^ and 1040 cm^−1^ observed for *E. uniflora* and peaks at 1206 cm^−1^ and 1045 cm^−1^ for *T. majus* are characteristic of stretching vibrations of the C-O and C-OH groups, associated with alcohol, ester, ether, and carboxylic acid groups present in phenolic compounds and flavonoids [50].

The FFS showed a peak at 2916 cm^−1^, corresponding to the C-H stretching of PVA and PVP [51], and at 1640 cm^−1^, characteristic of the C=C bond of PVA and PVP [52]. In C4, a peak was detected at 1084 cm^−1^, indicating the elongation of the C-O bond of the polymers [52].

Incorporating SEEU and SETM into the formulation resulted in changes in signal intensity and slight shifts in some wavelengths due to the interaction between molecules [50,52]. In addition, a characteristic broad band of hydroxyl hydrogen bonds was observed at 3290 cm^−1^ [49]. These connections can contribute to the maintenance of the polymeric structure by increasing the resistance of the material [53].

The interaction between molecules can also be observed in the mechanical properties (Table 7). The incorporation of SEEU and SETM resulted in a decrease in the tensile stress and elongation of the FFS. The integration of different actives of the crosslinking agents can cause a reduction in the resistance of the films due to the decrease in the binding of the polymeric structure [54].

The bending strength test without breaking can be used for product characterization and quality control [55]. It is possible to observe that all FFSs resisted the bending analysis, which can be explained by the properties of resistance and elasticity conferred by PVA [23].

**Figure 4:**
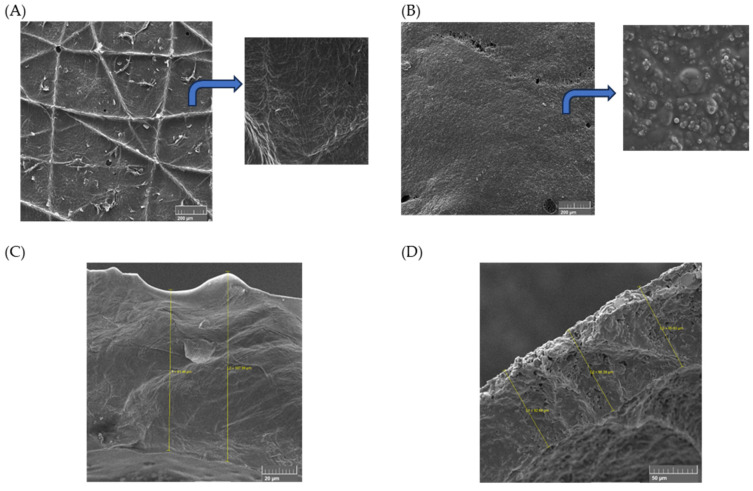
Surface Scanning electron microscopic images of (**A**) C4 and (**B**) F4; cryogenic cross-section of (**C**) C4 and (**D**) F4. Thickness measurements are also shown in images (**C**,**D**). The insets in (**A**,**B**) represent higher magnification (1 kx).

High values of WS were observed (≥88.31%) and attributed to PVA’s ability to absorb and retain high water amounts on its chains. This feature contributes a lot to maintaining structural moisture, which helps remove the film from the injured skin without damaging it [1]. On the other hand, water absorption can lead the fibre to swell, promoting a decrease in pores and reducing the water vapor permeation capacity [56]. Furthermore, the low WVTR values may be related to the low porosity of the films [57], demonstrated by scanning electron microscopy (SEM) (Figure 4).

Scanning electron images represent the surface and cross-sectional views of C4 and F4. The polymeric blend had basically formed a compact and homogeneous film, identifying miscible blending between PVA and PVP (see Figure 4C) [58]. The film presented the ability to mold itself to the skin, performing a perfect attachment by adhesion. The thickness of the film varied between 91 and 107 mm, as noted in Figure 4C. Structural modifications took place in the samples after adding the extracts, as seen in Figure 4B,D. Polymeric clusters were now present, making the surface rougher than the pristine blend. The film thickness appears to be constant (Figure 4D).

The incorporation of bioactive compounds into formulations aims to intensify biological functions, such as antioxidant and antimicrobial activities [59]. The FFS containing SEEU and SETM showed antimicrobial activity, whereas the control FFS did not show any inhibitory activity under the evaluated conditions (Table 8). The values presented correspond to the amount in mg mL^−1^ of FFS necessary for the antimicrobial effect, and F4 contains only 7.81% of SEEU and 3.90% of SETM, concentrations established from the synergism test using the checkerboard model.

In the initial phase of a skin infectious process, *S. aureus* is among the dominant organisms involved, whereas *E. coli* is found only in the final stages of the process when a chronic wound develops [60]. Fungi belonging to the genus *Candida* can cause infections ranging from those on the skin and mucous membranes to systemic infections, leading to the hematogenous dissemination of the yeast throughout the body [61].

Phenolic compounds can degrade the bacterial cell wall and cytoplasmic membrane, leading to the exudation of cell components, such as phosphates, ions, purines, and nucleic acids, or the introduction of substances harmful to metabolism, imparting an antibacterial action [62]. The SEEU and SETM used in this study had high concentrations of phenolic derivatives (93.28% and 98.17%, respectively), which could be related to their antimicrobial activity. Extracts with high concentrations of these compounds are promising candidates for the development of new microbial inhibitors [63].

Evaluation of the permeation profile helps to analyze the amount of permeated active compounds as a function of time. The evaluation of the optimized FFS loaded with SEEU and SETM (F4) showed practically zero permeation, and after 8 h under the evaluated conditions, only 0.97% of the bioactive compounds had permeated the skin.

Skin permeability is determined by the physicochemical characteristics of the formulation; therefore, certain factors, such as solubility, particle size, and electrical charge, can influence the process. In general, active compounds with solubility close to that of the skin have a higher rate of permeation [64]. In addition, smaller particles can agglomerate within the grooves present in the stratum corneum and facilitate transport to the deeper layers of the skin [65]. Positively charged particles can achieve greater permeation because the skin has a negative charge at physiological pH [66,67].

The FFS developed in this study aims at a local/topical antimicrobial effect; therefore, low permeation is desirable. However, in the in vitro evaluation, intact pig ear skin was used; the conditions differ from that of skin with an infectious process, where cellular barriers are compromised [68], resulting in altered permeation rates. Therefore, an in vivo study is necessary to estimate the concentration of bioactive molecules capable of reaching the systemic route and to prove the developed FFS’s safety.

Conventional treatment consists of applying ointments and creams. However, these have several limitations, as multiple daily applications are required, exposing the lesion to infectious agents [1]. Polymeric films based on biomaterials, on the other hand, can be absorbable, accelerating the tissue recovery process and making the patient return to their routine more quickly [48].

Additionally, this proposal aims to encourage the production of polymeric bio-dressings and to propose modern solutions for the pharmaceutical industry using environmentally friendly technologies and low-cost raw materials. Still, the prospection of this formulation is an alternative for treating topical infections, which has a widespread appeal aimed at those who do not wish to use synthetic drugs. The scope of this study was limited to the development of the FFS containing the SEEU and SETM combination. In future studies, the physicochemical stability and biodegradability should be evaluated.

## 3. Materials and Methods

### 3.1. Materials

PVA, PVP, and methylparaben were purchased from Dinâmica^®^ (São Paulo, Brazil), and imidazolidinyl urea was purchased from BIOTEC^®^ (São Paulo, Brazil). Neomycin was obtained from the Sovitá Ativos Company (São Paulo, Brazil). DPPH, ABTS, 2,4,6-Tris (2-pyridyl)-s-triazine, Trolox, Folin–Ciocalteu reagent, and ferric chloride were purchased from Sigma-Aldrich^®^ (St. Louis, MO, USA). Brain heart infusion (BHI) broth was obtained from Acumedia^®^ (Lansing, MI, USA). All other reagents were of analytical grade.

### 3.2. Preparation and Characterization of Extracts

#### 3.2.1. Sample Collection

Leaves of *E. uniflora* and *T. majus* were collected from the Horto de Plantas Medicinais at Universidade Paranaense, campus Umuarama (Paraná, Brazil; geographic coordinates 23°46′09.1″ S, 53°16′38.4″ W). The specimens were deposited in the Herbarium of Universidade Paranaense under numbers 12 and 59, respectively (SisGen number: A625A5A). The material was dried in a forced circulation oven at 40 °C until a constant weight was reached and then ground in a knife mill.

#### 3.2.2. Extract Preparation

The extracts were prepared by infusion of the ground samples in water at 90 °C for 6 h. Subsequently, the residue was filtered, and the infusion was treated with ethanol at an infusion:ethanol ratio of 1:3 for the precipitation of proteins and polysaccharides. The SETM and SEEU were obtained and concentrated in a rotary evaporator (Nova Ética, São Paulo, Brazil), lyophilized (JJ Científica, model LJJ02, São Paulo, Brazil), and stored in a freezer (−20 °C) until use [12].

#### 3.2.3. Phytochemical Characterization Using UHPLC/MS

Phytochemical characterization was performed using a Waters Acquity BEH C-18 UHPLC column (150 mm × 2.1 mm × 1.7 μm) with a flow rate of 560 μL min^−1^ and a column temperature of 60 °C. The mobile phases used were (A) water with 0.05% formic acid and (B) acetonitrile. The initial gradient consisted of a ratio (*v*/*v*) of 95:5 (A:B), which was linearly increased to 30:70 over 30 min. The gradient was then linearly increased to 5:95 in 0.1 min and maintained in this condition for 4 min. Subsequently, the gradient was immediately reversed to the initial condition for equilibration for 4 min.

For MS analysis, the quadrupole-time-of-flight system (Bruker, Q-TOFII^®^, Billerica, MA, USA) was used, with the following parameters: electroplate voltage of 4500 eV, nebulization gas flow of 3 L min^−1^, drying gas flow of 15 L min^−1^, and interface temperature of 250 °C. Nitrogen was used as the nebulizer and collision gas. Analyses were performed in positive and negative modes.

The chromatograms were evaluated using MetaboScape software (Bruker-USA^®^), where the obtained spectra were compared with various databases, such as the Bruker MetaboBASE^®^ Personal Library 3.0, Bruker HMDB Metabolite Library 2.0, and Bruker MetaboBASE^®^ Plant Library. Peaks with an intensity of at least 1000 were observed.

#### 3.2.4. Antioxidant Activity

For the combined antioxidant activity test, a sample with an initial concentration of 1000 µg mL^−1^ prepared with 333.3 µg mL^−1^ of SETM and 666.6 µg mL^−1^ of SEEU was used, corresponding to the amount used for the development of the FFS. To determine the theoretical/estimated value of the antioxidant effect, SETM and SEEU were evaluated individually at a concentration of 1000 µg mL^−1^. According to Velásquez et al. [69], when the experimental values are higher than the theoretical values, the effect of the combination is considered synergistic, and when the theoretical values are higher than the experimental values, the effect is antagonistic.

The scavenging capacity of DPPH and ABTS^•+^ radicals and the FRAP complex were evaluated in triplicate. For the DPPH analysis, a calibration curve was plotted with Trolox and fitted to the equation of the straight-line y = 0.5771x + 673.63 (R^2^ = 0.9968). The radical scavenging capacity was expressed in μmol L^−1^ Trolox equivalent, following a previously described methodology [70].

To evaluate the scavenging capacity of the ABTS^•+^ radical, a calibration curve was plotted using Trolox (line equation y = −0.2465x + 750.59; R^2^ = 0.9914). The ability to scavenge ABTS^•+^ radicals was presented in µmol of Trolox per gram of extract (µmol_Trolox_ g_ext_^−1^), following a methodology previously described [71].

To quantify the reducing potential of the FRAP complex, the equation y = 0.6188x − 96.833 (R^2^ = 0.9926) was obtained using ferrous sulfate, and the results were presented as µmol Fe^2+^ per g of extract (µmol_Fe_^2+^ g_ext_^−1^), as described by Santos et al. [72].

#### 3.2.5. Evaluation of Antimicrobial Activity Using the Checkerboard Model

The microorganisms used in the evaluation were the gram-positive *S. aureus* (ATCC 12026), gram-negative *E. coli* (ATCC 25922), and the yeast *C. albicans* (ATCC 10231). Microorganisms were cultured in BHI broth.

Standard suspensions containing 1.5 × 10^8^ CFU mL^−1^ were prepared in sterile saline for each microbial species. SEEU and SETM were solubilized in sterile water and evaluated separately and in combination at concentrations between 500 mg mL^−1^ and 0.488 mg mL^−1^. The experiments were performed in triplicate.

The microplates were incubated at 36 °C for 24 h (for bacteria) and at 25 °C for 48 h (for fungi). The MIC was defined as the lowest concentration of isolated and combined extracts capable of visibly inhibiting bacterial multiplication following the incubation period. The analysis of the results was performed by calculating the FICI, which is determined by Equation (1):

(1) $$FICI={FIC}_{A}+{FIC}_{B}=\frac{{MIC}_{A}\;in\;combination}{{MIC}_{A}\;separated}+\frac{{MIC}_{B}\;in\;combination}{{MIC}_{B}\;separated}$$

where A and B refer to SEEU and SETM, respectively.

According to Odds [73], FICI < 0.5 represents synergism, FICI > 4 represents antagonisms, and FICI between 0.5 and 4 is considered an absence of interaction.

### 3.3. Development and Characterization of the FFS

Colloidal FFSs were developed using a 2^2^ full factorial design with a central point to evaluate the influence of the independent variables (PVA and PVP concentration) on the development of the FFS loaded with SEEU and SETM. The complete factorial design matrices with coded and uncoded values for each factor are summarized in Table 9. The trials were randomized to reduce bias.

Methylparaben (0.1%) was heated in distilled water (q.s.p. 100%) to 80 °C. After natural cooling to room temperature, PVA (2.5, 3.5, or 4.5%) was dispersed in the solution through constant agitation for 2 h (Phase A). Simultaneously, PVP (2.5, 3.5, or 4.5%) was dispersed in 17.5% ethanol P.A. (Phase B), and the extracts (7.81% SEEU + 3.90% SETM) were dispersed in 12.5% ethanol 20° GL (Phase C). Phases B and C were magnetically stirred at room temperature for 2 h and added to Phase A through dripping. This was followed by the addition of imidazolidinyl urea solution (0.3%). After incorporation, agitation was maintained for 16 h at room temperature. The FFS was stored in a pump airless bottle equipped with a metered dose valve until physical–chemical characterization.

The control formulations (C1–C5), corresponding to each FFS, were prepared as described above, but without the addition of the extract.

#### 3.3.1. Organoleptic Evaluation

Five healthy men and five healthy women, aged between 20 and 40 years, with no history of skin diseases, were selected for organoleptic evaluation according to the protocol approved by the ethics committee for research involving human beings of Universidade Paranaense (5,832,179).

Approximately 0.3 mL of each FFS (amount corresponding to the volume delivered after each valve actuation) was applied to the back of the hand of the volunteer. The participants filled out a form indicating the drying time of the formulation (in minutes), adhesion, and appearance of the FFS. The film’s adhesion, appearance, clarity, shine, and transparency after drying were graded on scores. As parameters for bonding, 1—adhesive and 2—non-adhesive were used. As parameters for appearance, 1—shiny and transparent; 2—transparent, but without glare; 3—transparent, but flaky; 4—whitish film; 5—ruddy and shiny; 6—reddish but dull; and 7—reddish but scaly were used [74].

#### 3.3.2. pH

The pH of the FFS was determined in triplicate at 25 ± 1 °C using a calibrated digital potentiometer (Ionlab^®^, pHB 500, Araucária, Brazil).

#### 3.3.3. Volume of Solution Delivered to Each Actuation

The volume of the FFS delivered after each valve actuation was determined using an analytical balance (Gehaka^®^, AG-200, São Paulo, Brazil). Subsequently, the mean and standard deviation of 10 measurements were calculated. The volume of FFS delivered was calculated using Equation (2):

A_L_ = (W_t_ − W_0_) × D_n_ (2)

where A_L_: volume of solution delivered at each activation; W_t_: weight of the formulation after each actuation; W_0_: initial weight of the formulation, before activation; D_n_: density of the formulation [4].

#### 3.3.4. Viscosity

Viscosity was determined using a Brookfield Digital viscometer (QUIMIS^®^, Q860M26, Diadema, Brazil) equipped with a spindle No. 2, at 25 °C, in the range of 1 to 40 rpm [75].

### 3.4. Optimized FFS Characterization

#### 3.4.1. FTIR Spectroscopy

SEEU, SETM, and the FFS control (C4) and the FFS loaded with extracts (F4) were analyzed using FTIR (PerkinElmer, Waltham, MA, USA) and an attenuated total reflection accessory, at 25 °C, in the region of 4000 to 650 cm^−1^, at a resolution of 4 cm^−1^, for 9 scans [76].

#### 3.4.2. In Vitro Skin Permeation

In vitro skin permeation was evaluated using Franz-type diffusion cells mounted on porcine skin. The receiving compartment was filled with phosphate buffer (pH 7.4) and connected to a bath set at 32 ± 0.5 °C under constant agitation at 100 rpm. The total time of the assay was 8 h, with sampling times (400 µL) of 0.5, 1.0, 2.0, 4.0, 6.0, and 8.0 h, and volume replacement [77]. The total phenolic compound content was quantified using the Folin–Ciocalteu method [78]. A calibration curve was plotted (y = 14.269x + 65.544) using gallic acid, and the results were expressed in μg gallic acid equivalent/g extract (μg_EAG_ g_ext_^−1^). Control FFS exposed to the same permeation conditions was used.

#### 3.4.3. Mechanical Tests

Folding endurance was evaluated by repeatedly folding the film at the same place until it broke or folded up to 300 times, which was considered satisfactory [79]. This test was performed in triplicate.

Tensile and elongation tests: The mechanical strength was determined using a universal portable testing machine (Biopdi, São Carlos, Brazil) with a 5 kgf loaded cell and stretched at a constant rate of 5 mm s^−1^. The film’s thickness was measured using an analogue thickness gauge (Mitutoyo, No. 7301, Aurora, IL, USA). For each sample, the assay was performed in triplicate, and the average of the evaluation parameters was obtained [56].

#### 3.4.4. Barrier Properties

WS: Samples were cut into square shapes (2 cm^2^) and dried at 105 °C for 24 h before and after the solubilization period. After each drying period, the sample mass was measured using an analytical balance. WS was calculated using Equation (3):

(3) $$WS=\frac{{DM}_{0}-{DM}_{24}}{{DM}_{0}}\times 100$$

where DM_0_: weight of first dried material (before the solubilization); DM_24_: weight of dried material after 24 h of solubilization [80].

WVTR: Each sample (3.5 cm^2^) was fixed in an individual permeability cup containing 10 mL of distilled water, but the sample did not touch the water. The cup was kept in a desiccator containing previously dried silica gel. The set containing the test dish, buffer solution, and membrane was periodically weighed for 24 h, obtaining six points. The WVTR was calculated using Equation (4):

(4) $$WVTR=\frac{g\;.\;24}{t\;.\;a}$$

where *g*: mass loss (g); *t*: time (h); *a*: area (m^2^) [81].

#### 3.4.5. SEM

The cross-sectional microstructure and surface of the samples were investigated on a VEGA3 TESCAN scanning electron microscope (TESCAN, Libusina trída, Czech Republic) set to an accelerating voltage of 10 kV. Before analysis, the samples were dried in a desiccator and freeze-fractured using liquid nitrogen (N2). Afterwards, they were placed onto an aluminum pin stub. A thin layer of gold–palladium was deposited on the surfaces by an SC7620 sputtering device (Quorum Technologies, UK) for 180 s to avoid the surface charging effect.

#### 3.4.6. In Vitro Antimicrobial Activity

MIC was determined through serial microdilution in 96-well microplates, in triplicate, with BHI broth [82]. The test was performed against *S. aureus*, *E. coli*, and *C. albicans*. Concentrations ranging from 250 to 0.488 mg mL^−1^ of control FFS (C4) and loaded with SEEU and SETM (F4) were evaluated. The C4 formulation loaded with neomycin (conventional antibiotic for skin lesions) at a concentration of 5 mg mL^−1^ was used as the positive control (F4N). Plates were incubated at 36 °C for 24 h for bacteria and at 27 °C for 48 h for *C. albicans*.

### 3.5. Statistical Analysis

The analysis of variance (ANOVA) was used to evaluate the influence of the independent variables on the development of the FFS, at a confidence level of 95%. The software STATISTICA 13.0 (Statsoft^®^, USA) was employed in the statistical analysis.

## 4. Conclusions

The combination of SEEU and SETM increased the antioxidant effect through FRAP and ABTS^•+^ mechanisms, justifying the use of this combination for the development of an FFS. The physicochemical data suggest that formulation 4 (F4) prepared with 4.5% PVA, 4.5% PVP, 7.81% SEEU, and 3.90% SETM can be considered the optimized FFS. All formulations presented a pH similar to that of human skin and pseudoplastic flow behavior, but F3, F4, and F5 were more adhesive without the formation of scales and presented good malleability. F4 showed a shorter drying time than F3 and a higher resting viscosity than F3 and F5, ensuring better product stability. Through electronic scanning images, a constant thickness can be observed in the F4, promoting fixation by adhesion and, consequently, the ability to mold to the skin. Therefore, the antimicrobial effect of F4, in addition to the physicochemical results, indicate the potential of FFS for topical application for the differentiated treatment of cutaneous lesions.

## Figures and Tables

**Table 1:** Phytochemical characterization of soluble ethanolic fractions of *E. uniflora* (SEEU) using ultra-high-pressure liquid chromatography/mass spectrometry (UHPLC/MS).

Compound	*m*/*z*	Retention Time (min)	% *
Apigenin dimetil éter	311.101	25.78	12.42
Morin	299.021	19.15	9.23
Medicarpin 3-O-glucoside-6″-O-malonate	269.082	17.41	8.39
Astragalin	447.088	16.09	7.75
Luteolin 4′-O-glicoside	447.093	15.32	7.38
Spiraeoside	463.088	14.50	6.99
Naringin	579.172	12.56	6.05
Aspalathin	451.122	11.78	5.68
3-Ara-28-Glu hederagenin	927.491	11.61	5.59
Corymbosin	357.096	11.03	5.32
Nepetin	315.052	10.52	5.07
Scaposin	389.089	8.05	3.88
Myricetin	315.012	7.67	3.70
Robinetin	301.031	5.85	2.82
Synapol malate	353.070	4.22	2.03
Salicylic acid	137.024	3.80	1.83
Isosakuranin	447.132	3.36	1.62
Nevadensin	343.083	3.29	1.59
Kaempferide	299.057	3.21	1.55
Linolenic acid	279.232	2.31	1.11
Free flavonoids			45.56
Glycosylated flavonoids			43.86
Saponins			5.59
Cinnamic acid derivatives			2.03
Phenolic acids			1.83
Fatty acids			1.11

* Compound percentages were calculated based on the total number of identified compounds.

**Table 2:** Phytochemical characterization of soluble ethanolic fractions of *T. majus* (SETM) using UHPLC/MS.

Compound	*m*/*z*	Retention Time (min)	% *
Astragalin	447.088	16.09	12.81
Medicarpin 3-O-glucoside-6″-O-malonato	254.057	14.80	11.78
Quercetin 3-Glu-7-Rha	609.113	13.48	10.73
Naringin	579.172	12.56	10.00
Salicylic acid	137.026	8.19	6.52
Scaposin	389.089	8.05	6.41
Robinetin	301.031	5.85	4.66
Synapol malate	353.070	4.22	3.36
Pseudobaptigenin	281.049	3.93	3.13
Protocatechuic aldehyde	137.023	3.58	2.85
Chrysin	253.055	3.39	2.70
Neohesperidin	609.176	3.39	2.70
Isoferulic acid	193.001	3.38	2.69
Isosakuranin	447.132	3.36	2.67
Nevadensin	343.083	3.29	2.62
Naringenin 5,7-dimetil éter	299.092	3.23	2.57
Kaempferide	299.057	3.21	2.55
Quercitrin	445.101	3.13	2.49
4-Malonyl ononin	267.070	3.11	2.48
Genistein	269.048	3.10	2.47
Linolenic acid	279.232	2.31	1.84
Glycosylated flavonoids			55.65
Free flavonoids			27.10
Phenolic acids			9.37
Cinnamic acid derivatives			6.05
Fatty acids			1.84

* Compound percentages were calculated based on the total number of identified compounds.

**Table 3:** Antioxidative capacity of SEEU and SETM isolated and combined.

Method/Extract	*E. uniflora*	*T. majus*	E. uniflora + T. majus *			
			Theoretical Value	Experimental Value	%	Effect
DPPH (μM_Trolox_)	1123.95 ± 3.46	648.00 ± 39.41	964.33	751.61 ± 2.45	77.94	Antagonistic
FRAP (µmol_Fe_^2+^ g_ext_^−1^)	3983.25 ± 40.21	804.51 ± 63.60	2920.74	3312.05 ± 46.45	113.40	Synergistic
ABTS (µmol_Trolox_ g_ext_^−1^)	2692.05 ± 101.42	1094.87 ± 56.84	2157.49	6587.30 ± 191.37	305.32	Synergistic

* The combination was prepared using 333.33 µg mL^−1^ SETM and 666.66 µg mL^−1^ SEEU. Results are expressed as mean ± standard deviation (*n* = 3).

**Table 4:** Evaluation of the mean minimum inhibitory concentration (MIC) (mg mL^−1^) of SEEU and SETM alone and the synergistic effect of the combination on different microbial species.

MIC	*S. aureus*	*E. coli*	*C. albicans*
*E. uniflora*	7.81	15.62	15.62
*T. majus*	125.00	31.25	62.50
*E. uniflora* + *T. majus*	7.81 + 0.48	7.81 + 0.48	7.81 + 3.90
FICI	1.00	0.51	0.56

FICI = Inhibitory Fractional Concentration Index.

**Table 5:** Organoleptic analysis of control and film-forming system (FFS) loaded with SEEU and SETM.

FFS	Adhesion	Appearance	Drying Time ^a^ (min)
C1	1	3	16.00 ± 3.53
F1	1	7	54.20 ± 8.26
C2	1	1	21.90 ± 3.73
F2	1	7	24.90 ± 2.85
C3	1	1	26.00 ± 1.70
F3	1	6	44.10 ± 5.80
C4	1	1	33.40 ± 4.99
F4	1	6	36.00 ± 4.47
C5	1	1	29.90 ± 2.77
F5	1	6	31.00 ± 3.45

C1 = Control 1. F1 = Formulation 1. C2 = Control 2. F2 = Formulation 2. C3 = Control 3. F3 = Formulation 3. C4 = Control 4. F4 = Formulation 4. C5 = Control 5. F5 = Formulation 5. As parameters for adhesion, (1) adhesive and (2) non-adhesive were used. Appearance: (1) transparent and shiny; (2) transparent but without glare; (3) transparent but scaly; (4) whitish film; (5) ruddy and bright; (6) ruddy but dull; (7) ruddy but scaly. ^a^ Results are expressed as mean (*n* = 10) ± standard error.

**Table 6:** pH, volume of FFS delivered after each actuation, and viscosity of FFS prepared according to factorial design.

FFS	pH ^a^	Delivered Volume (mL)	Viscosity (mPa s) ^b^
C1	5.16 ± 0.06	0.43 ± 0.04	396.80
F1	4.37 ± 0.01	0.55 ± 0.01	1245.40
C2	4.81 ± 0.07	0.44 ± 0.03	634.60
F2	4.56 ± 0.01	0.45 ± 0.03	830.20
C3	5.30 ± 0.06	0.46 ± 0.03	538.20
F3	4.48 ± 0.04	0.56 ± 0.02	1895.10
C4	5.17 ± 0.08	0.43 ± 0.01	561.50
F4	4.46 ± 0.01	0.48 ± 0.01	2535.00
C5	4.98 ± 0.08	0.43 ± 0.04	580.50
F5	4.56 ± 0.07	0.49 ± 0.01	1573.50

C1 = Control 1. F1 = Formulation 1. C2 = Control 2. F2 = Formulation 2. C3 = Control 3. F3 = Formulation 3. C4 = Control 4. F4 = Formulation 4. C5 = Control 5. F5 = Formulation 5. ^a^ Results are expressed as mean ± standard deviation (*n* = 3). ^b^ Viscosity at 5 RPM.

**Table 7:** Mechanical and barrier properties of FFS control (C4) and loaded with SEEU and SETM (F4).

		C4	F4
Mechanical properties	Folding endurance (times)	>300	>300
	Tensile stress (MPa)	3.23 ± 0.35	0.66 ± 0.10
	Elongation at break (%)	11.10 ± 1.54	6.44 ± 0.45
	Modulus of elasticity (MPa)	135.51 ± 0.28	19.75 ± 0.02
Barrier properties	WS (%)	95.50 ± 1.34	88.31 ± 0.35
	WVTR(g h^−1^ m^−2^)	20.76 ± 0.75	21.01 ± 0.76

C4 = Control 4. F4 = Formulation 4. WS = Water solubility. WVTR = Water vapor transmission rate. Results are expressed as mean ± standard deviation (*n* = 3).

**Table 8:** Mean minimum inhibitory concentration (mg mL^−1^) of FFS against microbial species commonly found in topical infections.

FFS	*S. aureus*	*E. coli*	*C. albicans*
C4	−	−	−
F4	15.62	31.25	1.95
F4N	1.95	1.95	250.00

C4 = Control 4. F4 = Formulation 4. F4N = Control formulation 4 loaded with neomycin. (−) sign indicates no activity.

**Table 9:** Concentrations (%) and coded values used in the 2^2^ full factorial designs for developing the FFS loaded with SEEU and SETM.

Concentration	Formulation				
(%)	F1	F2	F3	F4	F5
PVA	2.5 (−1)	2.5 (−1)	4.5 (+1)	4.5 (+1)	3.5 (CP)
PVP	2.5 (−1)	4.5 (+1)	2.5 (−1)	4.5 (+1)	3.5 (CP)

F1 = Formulation 1. F2 = Formulation 2. F3 = Formulation 3. F4 = Formulation 4. F5 = Formulation 5. CP = Central point.

## Data Availability

Data are contained within the article.
